# The impact of recreational marijuana commercialization on traumatic injury

**DOI:** 10.1186/s40621-019-0180-4

**Published:** 2019-02-04

**Authors:** Christine Chung, Kristin Salottolo, Allen Tanner, Matthew M. Carrick, Robert Madayag, Gina Berg, Mark Lieser, David Bar-Or

**Affiliations:** 1 Trauma Research Department, Swedish Medical Center, Englewood, CO 80113 USA; 2 Trauma Research Department, Penrose-St Francis Health Services, Colorado Springs, CO USA; 3 Trauma Research Department, Medical City Plano, Plano, TX USA; 4 Trauma Research Department, St. Anthony Hospital, Lakewood, CO USA; 5 Trauma Research Department, Wesley Medical Center, Wichita, KS USA; 6 Trauma Research Department, Research Medical Center, MO, Kansas City, US USA

**Keywords:** Marijuana, Legalization, Activation, Urine drug screen

## Abstract

**Background:**

The medical legalization of marijuana has been shown to result in an increased risk of motor vehicle injuries. In Colorado, commercialization of recreational marijuana (initiation of retail sales) occurred on January 1, 2014, resulting in the rapid proliferation of its availability. The objective of this study was to determine whether the proportion of injured patients testing positive for marijuana and other drugs changed two years before and two years after commercialization of recreational marijuana in Colorado.

**Methods:**

This retrospective multi-institutional cohort study included all patients admitted with a traumatic injury to six trauma centers (three centers in Colorado and three centers in states without permissive marijuana laws) from 2012 to 2015. The primary outcome was the rate (%) of a positive urine drug screen (UDS) for marijuana. Generalized linear regression models were used to examine the rate of change over time in the pre-commercialization period relative to the post- commercialization period (via an interaction effect), separately for states with and without recreational marijuana legalization.

**Results:**

There were 40,591 trauma admissions. In Colorado, the rate of marijuana detected with UDS decreased over time pre-commercialization; this trend reversed post-commercialization, when marijuana detection rates increased significantly over time (interaction *p* = 0.004). At non-Colorado hospitals, the rate over time of marijuana detection was significantly reduced post-commercialization relative to the pre-commercialization period (*p* <  0.001).

**Conclusion:**

There was an overall increased rate over time of marijuana detected among trauma patients at Colorado hospitals but not at non-Colorado hospitals, suggesting an increased use of marijuana or an increased risk of injury while using marijuana following the commercialization of recreational marijuana in Colorado.

**Electronic supplementary material:**

The online version of this article (10.1186/s40621-019-0180-4) contains supplementary material, which is available to authorized users.

## Background

Cannabis (marijuana) is a commonly used drug, with increased availability and utilization since its legalization for both medical and recreational use in the United States (Cherpitel et al., 2017; Monte et al., 2015). Although the use of cannabis remains federally illegal, as of 1/1/2019 the medical use of cannabis is legal in 33 states and the recreational use of cannabis is legal in ten states and the District of Columbia (National Conference of State Legislatures, 2018). In the state of Colorado, medical legalization of marijuana began in 2000, and recreational legalization occurred on 11/16/2012 via constitutional amendment. Colorado was the first state to initiate commercial (retail) sale of recreational marijuana to the general public on 1/1/2014.

Traumatic injury, defined as any physical injury of sudden onset requiring immediate medical attention, is the leading cause of death among persons < 45 years old in the United States, and results in nearly three million hospitalizations each year. (Centers for Disease Control and Prevention, 2018) Marijuana use and intoxication has been shown to increase the risk of traumatic injury, especially fatal motor vehicle crashes (MVCs) (Asbridge et al., 2012; Asbridge et al., 2014; Aydelotte et al., 2017; Li et al., 2012; Salomonsen-Sautel et al., 2014; Couper & Peterson, 2014). Other research on injury related diagnoses have demonstrated an increased risk of burn injuries (Jehle Jr. et al., 2015), and non-traffic injuries with medical legalization of marijuana, particularly falls for older adults (Barrio et al., 2012). There is limited research examining the association between risk of injury with recreational marijuana legalization. A study by Aydelotte et al. examined the effect of recreational marijuana legalization on injury by analyzing year-over-year changes in MVC fatality rates before and after recreational legalization of marijuana in Colorado and Washington, the first two states to legalize marijuana, compared to eight control states. The authors report similar MVC crash fatality rates for Washington and Colorado compared to control states (Aydelotte et al., 2017).

Overall, however, there is a paucity of epidemiological data on the role of recreational marijuana legalization on traumatic injury. The objective of this study was to determine whether the proportion of injured patients testing positive for marijuana and other drugs changed two years before and two years after commercialization of recreational marijuana in Colorado.

## Methods

### Setting, study design, and participants

This multi-institutional, retrospective study was performed by the Injury Outcomes Network, a collaborative research network of six community based, level I trauma centers: three trauma centers are located in Colorado (Swedish Medical Center, St. Anthony Hospital, Penrose Hospital), and three trauma centers are located in states without recreational or medical marijuana laws (Medical City Plano, Plano, TX, Research Medical Center, Kansas City, MO, and Wesley Medical Center, Wichita, KS). The study included all consecutive hospital admissions with traumatic injury regardless of age, two years before (1/1/2012–12/31/2013) and two years after (1/1/2014–12/31/2015) the commercialization of recreational marijuana in Colorado. Patients were identified by the hospital trauma registry (Traumabase® or Digital Innovations, Inc.). This study was IRB approved at all participating centers and was granted a waiver of consent and HIPAA authorization.

### Study variables

The following demographic, clinical, and outcome characteristics were obtained from the trauma registries: trauma activation level (full = highest activation level, partial, not activated); admission date; whether a toxicology urine drug screen (UDS) was performed (yes/no); UDS test results (positive or negative for each drug in a multi-drug panel that includes marijuana [tetrahydrocannabinol, THC], amphetamines, barbiturates, benzodiazepines, cocaine, PCP, and opiates); age, years; gender (M/F); race (coded as White and non-White; ethnicity was not examined); mechanism of injury (blunt vs. penetrating); cause of injury (MVC, fall, sports related, gunshot wound/assault, other injury); injury severity score (ISS); Glasgow coma score on admission (3-8, 9-15); systolic blood pressure on admission (< 90 mmHg or ≥ 90 mmHg); in-hospital mortality; ICU admission.

### Outcome measures

The primary outcome was the percent of patients with a positive UDS finding of marijuana (THC). We examined differences over time across eight six-month study periods (where period 1 = 1/1/2012–6/30/2012 and period 8 = 7/1/2015–12/31/2015) for the percent of patients with marijuana detected on UDS. We also examined differences over time across eight six-month study periods for the percent of patients who had a positive UDS test for other drugs (amphetamines, barbiturates, benzodiazepines, cocaine, PCP, opiates). The rate of change over time was compared for the pre-commercialization period (four six-month periods from 1/1/2012–12/31/2013) relative to the post-commercialization period (four six-month periods from 1/1/2014–12/31/2015).

### Statistical analyses

Generalized linear regression models were used to examine the rate of change over time in the pre-commercialization period relative to the post- commercialization period via an interaction effect, adjusting for changes in the proportion of patients who received UDS testing over time. The model was used to examine the interaction between commercialization period (pre/post), and time period (across eight six-month periods). A significant interaction demonstrates that the rate of a positive UDS test result (the slope) differed before vs. after recreational marijuana commercialization. The model did not adjust for other covariates.

The analyses were performed separately for states with and without recreational legalization, in three populations: 1. All admissions; 2. Full trauma team activations; 3. MVC injuries. The analyses are presented for patients who required full trauma team activation only; results for all admissions and those with MVC injuries can be found in Additional file 1: Figures S1–S4. Trauma activations had the highest rate of UDS testing, and results were representative of the entire population and those with MVC injuries.

Pearson chi-square tests and student’s t-tests were used to compare demographics, injury characteristics, and outcomes. All statistical analyses were two-tailed with significance defined as *p* value < 0.05 and were conducted using SAS 9.4 (SAS Institute Inc., Cary, NC).

## Results

### Patient characteristics

**Fig. 1:**
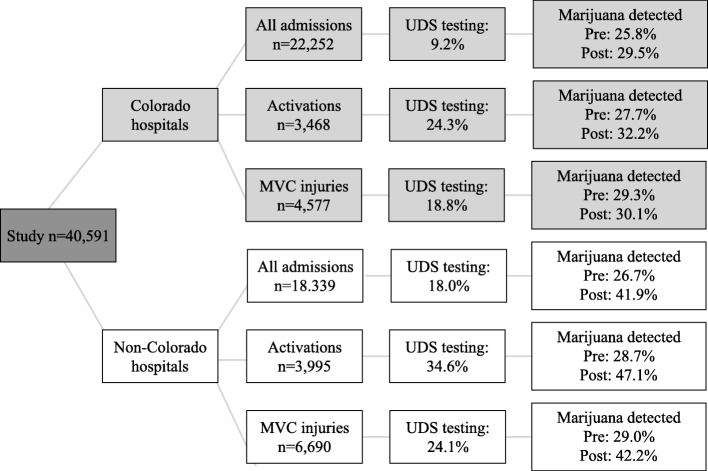
Population distribution and rate (%) of UDS testing. UDS, urine drug screen; MVC, motor vehicle crash. Activation refers to a full trauma team activation (highest level). Pre, 2012–2013. Post, 2014–2015

There were 40,591 injured patients admitted over the study period, including 7463 full trauma activations and 11,267 patients who suffered an MVC injury (Fig. 1). There were significant differences in the patient populations seen at Colorado hospitals compared to hospitals outside Colorado (Table 1). The Colorado population was older, predominantly White, with less severe injuries. There were no clinically relevant changes in the composition of the populations before and after commercialization, for Colorado hospitals and for non-Colorado hospitals.

**Table 1:** Differences in Colorado vs. non-Colorado hospitals

Covariate	Colorado (*n* = 22,252)	Non-Colorado (*n* = 18,339)	*P* value
Age, mean	57.71y	47.57y	< 0.001
Male sex, %	55.6%	59.3%	< 0.001
Non-White race, %	18.0%	25.2%	< 0.001
Glasgow Coma score 3–8	5.4%	8.9%	< 0.001
Injury severity score, mean	9.68	9.92	0.007
SBP < 90 mmHg	2.7%	3.2%	0.008
Penetrating Mechanism	3.8%	7.3%	< 0.001
Cause of injury			< 0.001
Motor vehicle crash	20.6%	36.5%	
Fall	55.6%	40.6%	
Sports related	12.8%	3.6%	
Assault/Stab/GSW	5.9%	7.8%	
Other causes	5.2%	11.6%	
Full activation	15.6%	21.8%	< 0.001
ICU admission	31.7%	38.8%	< 0.001
Mortality	3.0%	4.5%	< 0.001

Pre, 2012–2013. Post, 2014–2015. *SBP* systolic blood pressure, *GSW* gunshot wound, *ICU* intensive care unit

There were very few differences in patient characteristics who were drug tested pre- vs. post-commercialization (Table 2). At non-Colorado hospitals, there were more penetrating mechanisms (stabbing, gunshot wounds) in patients who had UDS testing in the post-commercialization period compared to the pre-commercialization period. In Colorado, there were fewer activations in patients with UDS testing post-commercialization, whereas in non-Colorado states, there were more activations in patients with UDS testing post-commercialization compared to the pre-commercialization period.

**Table 2:** Differences in patients who had urine drug screen testing pre vs. post commercialization in Colorado, by hospital location

	Colorado hospitals			Non-Colorado hospitals		
Covariate (%)	UDS performed Pre (*n* = 1027)	UDS performed Post (*n* = 1013)	*P* value	UDS performed Pre (*n* = 1786)	UDS performed Post (*n* = 1507)	*P* value
Age (years), mean	42.2	43.9	**0.03**	41.0	39.8	0.06
Male sex	69.9%	72.2%	0.26	67.4%	69.7%	0.15
Non-White race	28.1%	24.7%	0.08	23.7%	37.0%	**< 0.001**
Glasgow Coma score 3–8	17.2%	16.6%	0.74	17.7%	19.6%	0.16
Injury severity score, mean	12.8	12.7	0.87	11.5	11.4	0.95
SBP < 90 mmHg	5.0%	4.4%	0.53	3.5%	4.7%	0.08
Penetrating mechanism	7.2%	7.6%	0.85	8.7%	11.4%	**< 0.001**
Cause of injury			0.36			**< 0.001**
Motor vehicle crash	43.9%	40.4%		48.6%	49.4%	
Fall	31.3%	33.0%		24.6%	20.6%	
Sports related	7.6%	8.8%		3.5%	2.1%	
Assault/Stab/GSW	14.8%	14.5%		11.5%	15.9%	
Other	2.4%	3.4%		11.8%	11.9%	
Full activation	44.3%	38.3%	**< 0.001**	38.8%	45.7%	**< 0.001**
ICU admission	53.4%	57.2%	0.08	53.6%	55.5%	0.27
Mortality	4.8%	4.7%	0.97	5.9%	5.1%	0.34

Pre-commercialization, 2012–2013. Post-commercialization, 2014–2015. *SBP* systolic blood pressure, *GSW* gunshot wound, *ICU* intensive care unit. Bolding denotes p values that met the alpha < 0.05 significance level

The percent of patients with marijuana detected pre- vs. post- commercialization is shown in Fig. 1. There were demographic differences in patients testing positive for marijuana before vs. after commercialization (Table 3). At Colorado hospitals, patients testing positive for marijuana in the post-commercialization period were slightly older and were more frequently White, compared to the pre-commercialization period. However, at non-Colorado hospitals, patients testing positive for marijuana in the post-commercialization period were less frequently White.

**Table 3:** Differences in positive marijuana detection pre vs. post commercialization in Colorado, by hospital location

	Colorado hospitals			Non-Colorado hospitals		
Covariate, %	Marijuana detected Pre (*n* = 265)	Marijuana detected Post (*n* = 299)	*P* value	Marijuana detected Pre (*n* = 476)	Marijuana detected Post (*n* = 632)	*P* value
Age (years), mean	34.1	36.6	**0.04**	32.4	33.1	0.35
Male sex	74.0%	78.9%	0.16	76.1%	78.0%	0.44
Non-White race	34.3%	26.1%	**0.03**	40.8%	55.7%	**< 0.001**
Glasgow Coma score 3–8	16.1%	15.1%	0.74	13.7%	16.5%	0.20
Injury severity score, mean	12.2	11.5	0.44	10.1	11.5	**0.02**
SBP < 90 mmHg	4.6%	4.1%	0.76	3.8%	5.6%	0.17
Penetrating Mechanism	7.6%	9.0%	0.70	16.2%	16.9%	0.09
Cause of injury			0.13			0.14
Motor vehicle crash	49.8%	41.4%		52.9%	49.7%	
Fall	20.4%	26.8%		12.8%	13.6%	
Sports related	10.9%	9.4%		2.9%	1.4%	
Assault/Stab/GSW	15.9%	17.4%		20.2%	25.2%	
Other	3.0%	5.4%		11.1%	10.1%	
Full activation	47.6%	41.8	0.16	41.8%	51.3%	**< 0.001**
ICU admission	45.7%	53.5%	0.77	47.9%	47.0%	0.77
Mortality	2.6%	1.7%	0.43	3.4%	2.2%	0.24

Pre-commercialization, 2012–2013. Post- commercialization, 2014–2015. *SBP* systolic blood pressure, *GSW* gunshot wound, *ICU* intensive care unit. Bolding denotes *p* values that met the alpha < 0.05 significance level

### Results, Colorado

**Fig. 2:**
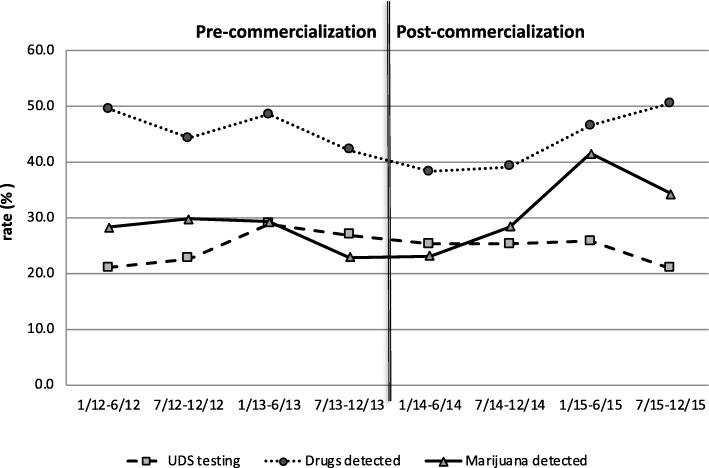
Colorado trends in urine drug screen (UDS, %) detection of marijuana and other drugs over time across eight six-month periods from 2012 to 2015, before and after commercialization of recreational marijuana

The rate of marijuana detected in the activated population is presented in Fig. 2. In activated patients, there was a significant interaction effect for marijuana use: commercialization of recreational marijuana was associated with an increased rate of marijuana detection (interaction *p* < 0.001). Specifically, the rate of detecting marijuana increased post-commercialization, whereas it was decreasing in the pre-commercialization period. The proportions in the overall population and the subset with an MVC injury were similar and are presented in Additional file 1: Figures S1 and S2.

There was also a significant interaction effect for the model examining other drug use, interaction *p* < 0.001. The rate of detecting other drugs increased post-commercialization relative to pre-commercialization, where it was decreasing over time (Fig. 2). The results in the overall population and the subset with an MVC injury were identical to the activation population (interaction *p* < 0.001); these rates are displayed in Additional file 1: Figures S1 and S2.

### Results, non-Colorado hospitals

**Fig. 3:**
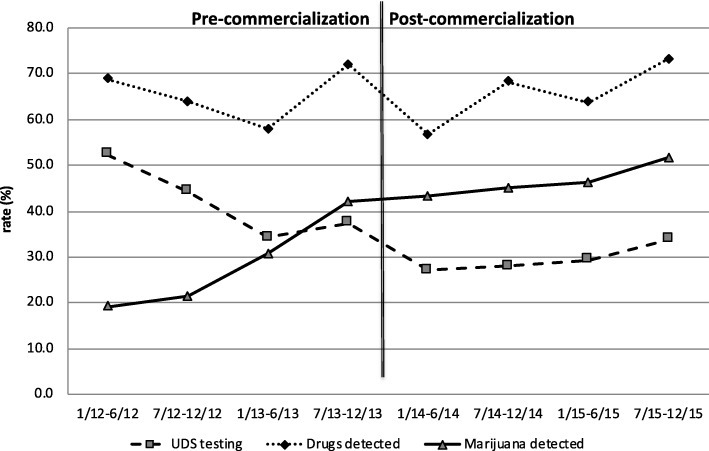
Non-Colorado state trends in urine drug screen (UDS, %) detection of marijuana and other drugs over time across eight six-month periods from 2012 to 2015, before and after commercialization of recreational marijuana

The rate of marijuana detected in the activated population is presented in Fig. 3 in states without recreational or medical marijuana laws. Despite an increase in marijuana detection over the study period, the model demonstrated a significant interaction effect for marijuana use (interaction p < 0.001). Specifically, the rate of marijuana detection was increasing over time in the pre-commercialization period, relative to the leveling off that was observed in the post-commercialization period. The results in the overall population and the subset with an MVC injury were identical (interaction *p* < 0.001); rates are displayed in Additional file 1: Figures S3 and S4.

There was also a significant interaction effect for the model examining other drug use (interaction *p* = 0.02). Here, detection of other drugs was unchanged in the pre-commercialization period, relative to the increased detection of other drugs that was observed in the post-commercialization period (Fig. 3). The results for other drug use in the overall population and the subset with an MVC injury are shown in Additional file 1: Figures S3 and S4. In these populations, there was no interaction effect for other drugs; the rate of a detecting other drugs was unchanged in the pre-commercialization period relative to the post-commercialization period, overall (interaction *p* = 0.30) and in the subset with an MVC injury (interaction *p* = 0.63).

## Discussion

The results of our study demonstrate that in the two years after recreational marijuana was commercialized in Colorado in 2014, there was an increasing rate of detecting marijuana for patients presenting to Colorado hospitals with a traumatic injury, relative to the pre-commercialization period. Additionally, there was a significant increased rate of detecting other drugs post-commercialization, suggesting that commercialization of marijuana may have contributed to an increased use of other drugs preceding the traumatic injury. Conversely, relative to the pre-commercialization period, the rate of detecting marijuana was significantly reduced in the post-commercialization period at non-Colorado hospitals.

Taken together, these results may indicate that commercialization of recreational marijuana is associated with an increased use of marijuana or an increased risk of traumatic injury while using marijuana. Although the results of this study were statistically significant, there is a need for further investigation, as causality is not established.

With acute use, marijuana intoxication is associated with impairment of attention and concentration, impulse control, planning, decision-making, response times, visio-spatial selective attention, short-term memory, and motor control (Broyd et al., 2016). In chronic use, there is persistent impairment of attention, verbal and working memory, decision-making, and executive function (Broyd et al., 2016). Several studies have demonstrated that marijuana use is associated with increased risk of traumatic injury, especially more severe injuries. In a systematic review and meta-analysis, Asbridge et al. found that driving under the influence of cannabis was associated with almost doubling the risk for being involved in a MVC that resulted in serious injury or death (Asbridge et al., 2012). Another meta-analysis of nine epidemiologic studies identified patients using marijuana were more than twice as likely to be involved in a car crash, and the risk of crash involvement increased in a dose-responsive manner with the concentration of THC detected in urine and frequency of self-reported marijuana use (Li et al., 2012).

While there are numerous studies examining the effect of marijuana use on injury, few studies have examined the effect of changes in marijuana laws on injury. Four studies have examined trends in fatal MVCs before and after changes in marijuana laws using the Fatality Analysis Reporting System (FARS) (Aydelotte et al., 2017; Salomonsen-Sautel et al., 2014; Mark Anderson BH and Rees, 2013; Santaella-Tenorio et al., 2017). Salomonsen-Sautel et al. examined temporal changes in the proportion of drivers in a fatal MVC who were alcohol impaired (> 0.08 g/dl) and who were marijuana positive before and after medical marijuana legalization (1994–2011) (Salomonsen-Sautel et al., 2014). The proportion of drivers in fatal MVCs who were marijuana positive in Colorado was decreasing during the pre-legalization period, and similar changes were not seen in cases of alcohol impairment. During the post-legalization period, there was a higher increasing trend in the proportion of drivers in fatal MVCs who were marijuana positive in Colorado than in states with restrictive medical marijuana laws. On the contrary, two studies utilizing the FARS reported that states with medical marijuana laws reported a reduction in the rates of traffic fatalities. One of these studies also found the presence of dispensaries to be associated with a reduction in traffic fatalities (Santaella-Tenorio et al., 2017). In the other study, the reduction in traffic fatalities was thought to be related to an increase in marijuana use leading to a decrease in alcohol consumption (substitution), partially explaining the reduction in alcohol-related fatalities observed in states with permissive medical marijuana laws (Mark Anderson BH and Rees, 2013). The study by Aydelotte and colleagues is the only study to examine temporal trends following changes in recreational legalization laws. Crash fatality rates were no different in Colorado and Washington compared to states without recreational marijuana legalization (Aydelotte et al., 2017). In our study, the trend of marijuana detection at Colorado hospitals appeared to decrease pre-commercialization, with an increasing trend of marijuana detection in the post- commercialization period. Similar findings were reported in patients with MVC injuries.

The prevalence of UDS testing and positive test results were higher in non-Colorado hospitals than Colorado hospitals. This is likely a result of the populations: trauma patients admitted to the Colorado hospitals were older and more likely to have a fall injury, whereas patients admitted to the non-Colorado hospitals were younger, more severely injured, and involved in MVCs. In general, selective UDS testing is usually associated with a higher index of suspicion, while broader UDS testing is associated with less frequent positive findings. If the rate of testing decreases over time, then one would expect the proportion of positive test results to increase. In Colorado, the rate of UDS testing was consistent across the study period. However, in states with restrictive marijuana laws, the rate of UDS testing significantly decreased during the study period. The findings that there was a lower *rate* of positive marijuana results in the post-legalization period is thus even more significant because it would be expected that the rate of positive findings would increase with decreasing (selective) UDS testing. We adjusted for changes in the UDS testing rate over time to help lessen this possible selection bias/overestimation.

There are several study limitations. First and foremost, testing positive for marijuana is not incontrovertible evidence for intoxication at the time of or the cause of injury. While the half-life of THC is relatively short (approximately 30 min), the amount, route, and concentration all affect how long marijuana is detectable in urine. THC and its metabolites are detectable in urine for three days in occasional users (< 4 times/week), and greater than ten days in daily chronic users (Moeller et al., 2017). Second, marijuana and drug use were defined by UDS findings only. We were unable to review records for self-reported marijuana use and it is not recorded electronically in the trauma registry. However, Asbridge et al. published a study examining 860 drivers in Canada with MVC injury over two years, examining blood samples and self-reported marijuana use; positive detection of marijuana was associated with a four-fold increased odds of collision, whereas there was no significant association when relying just on self-report measures (Asbridge et al., 2014). Third, there were some differences in the patients who were tested pre- and post-commercialization, in both Colorado and non-Colorado hospitals, that were not accounted for in the analyses and may have influenced results. Fourth, we used separate models for Colorado hospitals and non-Colorado hospitals, rather than a difference-in-differences approach where all data could be modeled together (Wing et al., 2018). The difference-in-differences approach has been used in this context previously (Aydelotte et al., 2017; Bradford and Bradford, 2016; Pacula et al., 2015), and is used for policy decisions. We were unable to use this analytical approach because our data violated the common trends assumption, that the baseline pre-legalization period rates of change are not significantly different for Colorado hospitals and non-Colorado hospitals. Lastly, our study determined the effect of commercialization of recreational marijuana on the rate of marijuana detection among trauma patients. We do not know the effect of how the political campaign before the vote, and the period after the legalization vote and prior to commercialization, might have influenced use.

## Conclusions

In Colorado, there was an increased rate of marijuana detected over time after the commercialization of recreational marijuana relative to the pre-commercialization period, which was not observed at non-Colorado hospitals. These results suggest that there is an increased use of marijuana and other drugs or an increased risk of injury in patients using marijuana and other drugs, after the commercialization of recreational marijuana in Colorado. Although the use of cannabis remains federally illegal, as of this publication ten states have legalized recreational marijuana, with more states potentially following suit. Knowing what effect recreational marijuana legalization has on injury, a leading cause of death for Americans less than 44 years of age, may encourage additional research and also support policy, program, and education interventions. A larger, nationwide evaluation of recreational marijuana legalization is warranted since these additional nine states have commercialized recreational marijuana since Colorado’s implementation in 2014.

## Additional file

Additional file 1: Trends in urine drug screen (UDS, %) detection of marijuana and other drugs over time across eight six-month periods from 2012-2015, before and after commercialization of recreational marijuana. **Figure S1.** Colorado, all admissions; **Figure S2.** Colorado, MVC injury; **Figure S3.** Non-Colorado hospitals, all admissions; **Figure S4.** Non-Colorado hospitals, MVC injury. (PDF 191 kb)

## Abbreviations

AIS: Abbreviated injury scale
GSW: Gunshot wound
ICU: Intensive care unit
ISS: Injury severity score
MVC: Motor vehicle crash
SBP: Systolic blood pressure
THC: Tetrahydrocannabinol
UDS: Urine drug screen
