# Supplementary material for: The impact of recreational marijuana commercialization on traumatic injury
Source: Inj Epidemiol. 2019 Feb 4;6:3. doi: 10.1186/s40621-019-0180-4 (PMC6360194; doi:10.1186/s40621-019-0180-4)
Supplement: Supplementary file 1 — Trends in urine drug screen (UDS, %) detection of marijuana and other drugs over time across eight six-month periods from 2012-2015, before and after commercialization of recreational marijuana. Figure S1. Colorado, all admissions; Figure S2. Colorado, MVC injury; Figure S3. Non-Colorado hospitals, all admissions; Figure S4. Non-Colorado hospitals, MVC injury. (PDF 191 kb) [file 40621_2019_180_MOESM1_ESM.pdf]

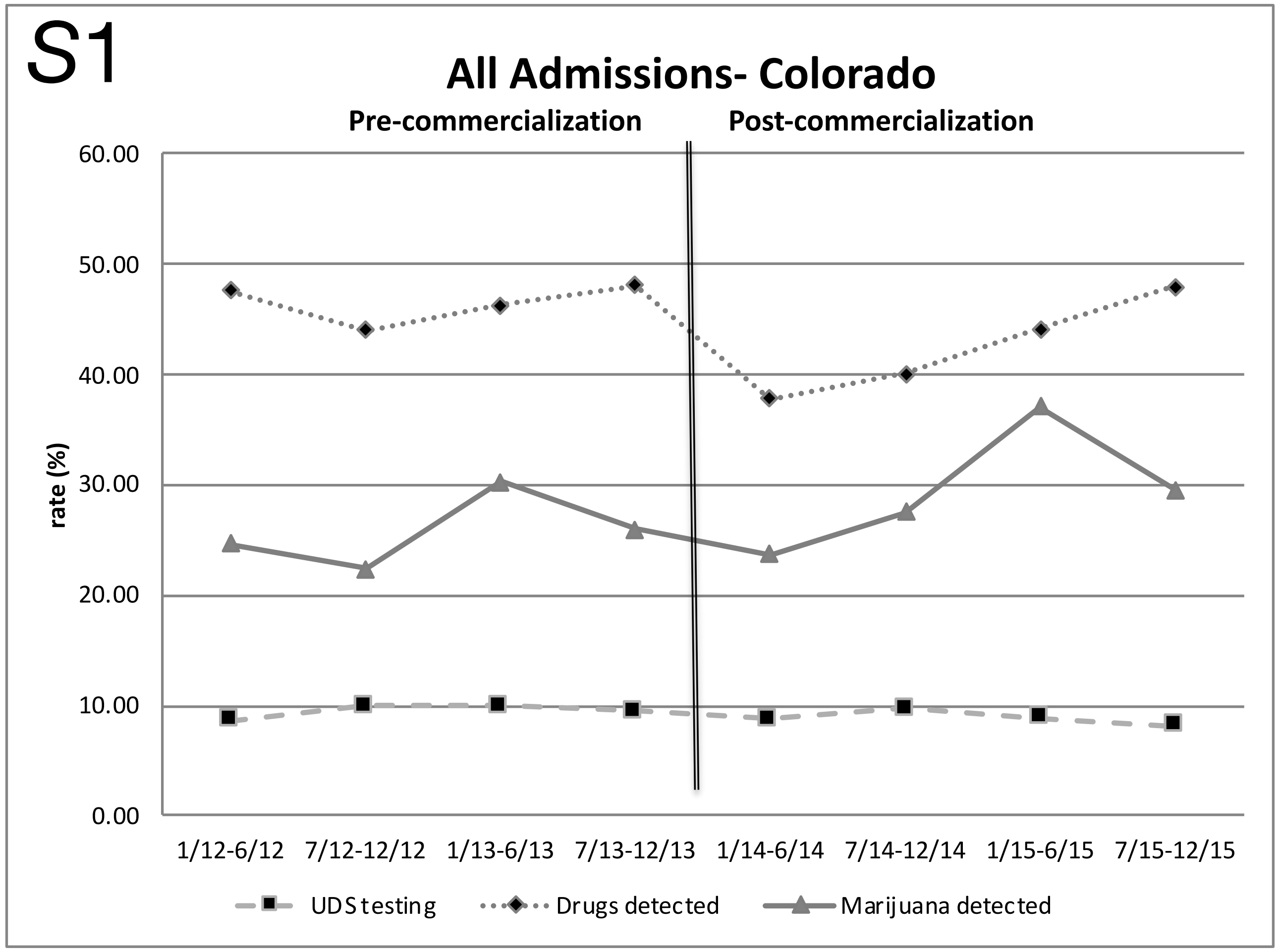
S1
All Admissions- Colorado
Pre-commercialization
Post-commercialization
rate (%)
0.00
10.00
20.00
30.00
40.00
50.00
60.00
1/12-6/12
7/12-12/12
1/13-6/13
7/13-12/13
1/14-6/14
7/14-12/14
1/15-6/15
7/15-12/15
UDS testing
Drugs detected
Marijuana detected

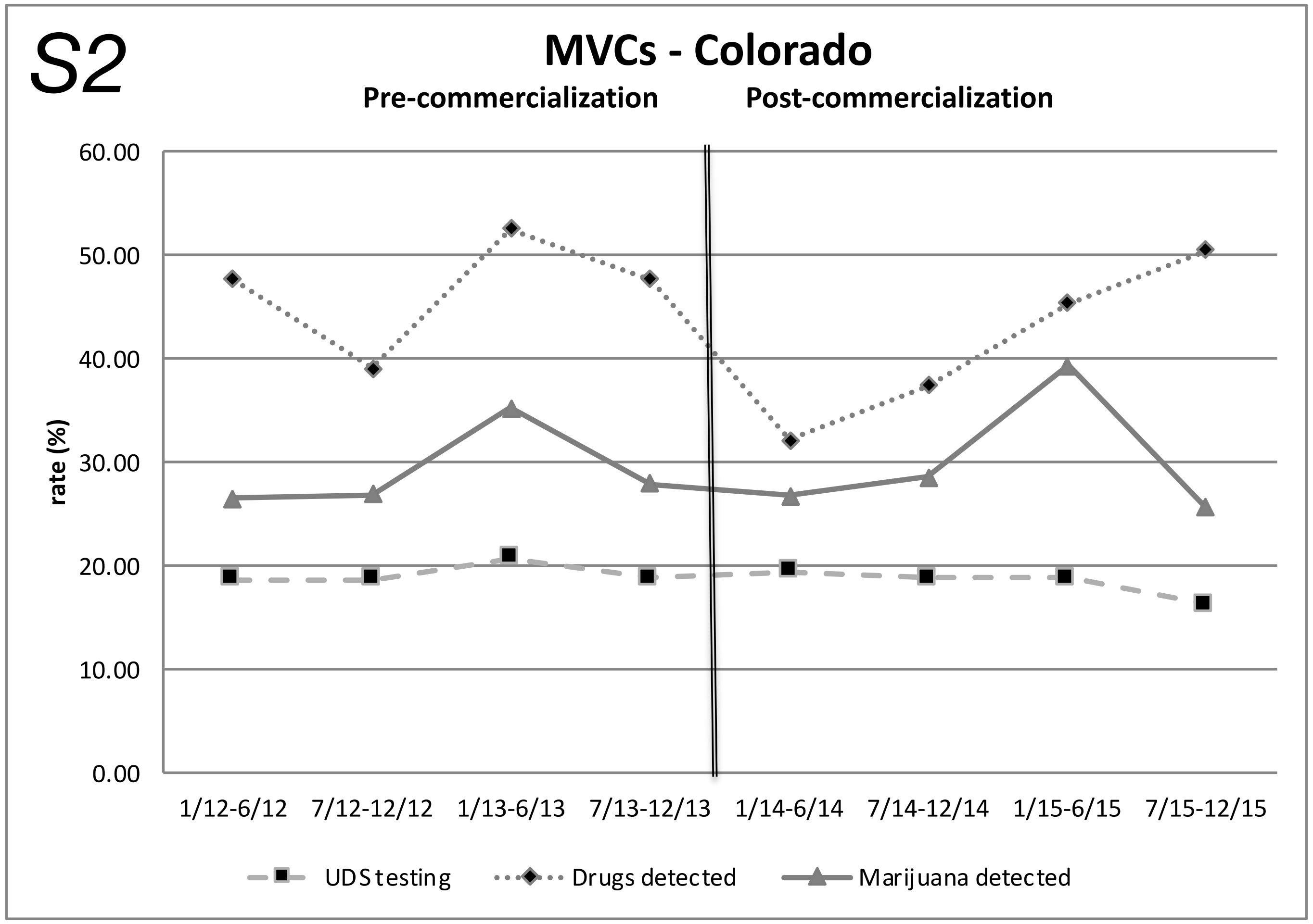
S2
MVCs - Colorado
Pre-commercialization
Post-commercialization
rate (%)
0.00
10.00
20.00
30.00
40.00
50.00
60.00
1/12-6/12
7/12-12/12
1/13-6/13
7/13-12/13
1/14-6/14
7/14-12/14
1/15-6/15
7/15-12/15
UDS testing
Drugs detected
Marijuana detected

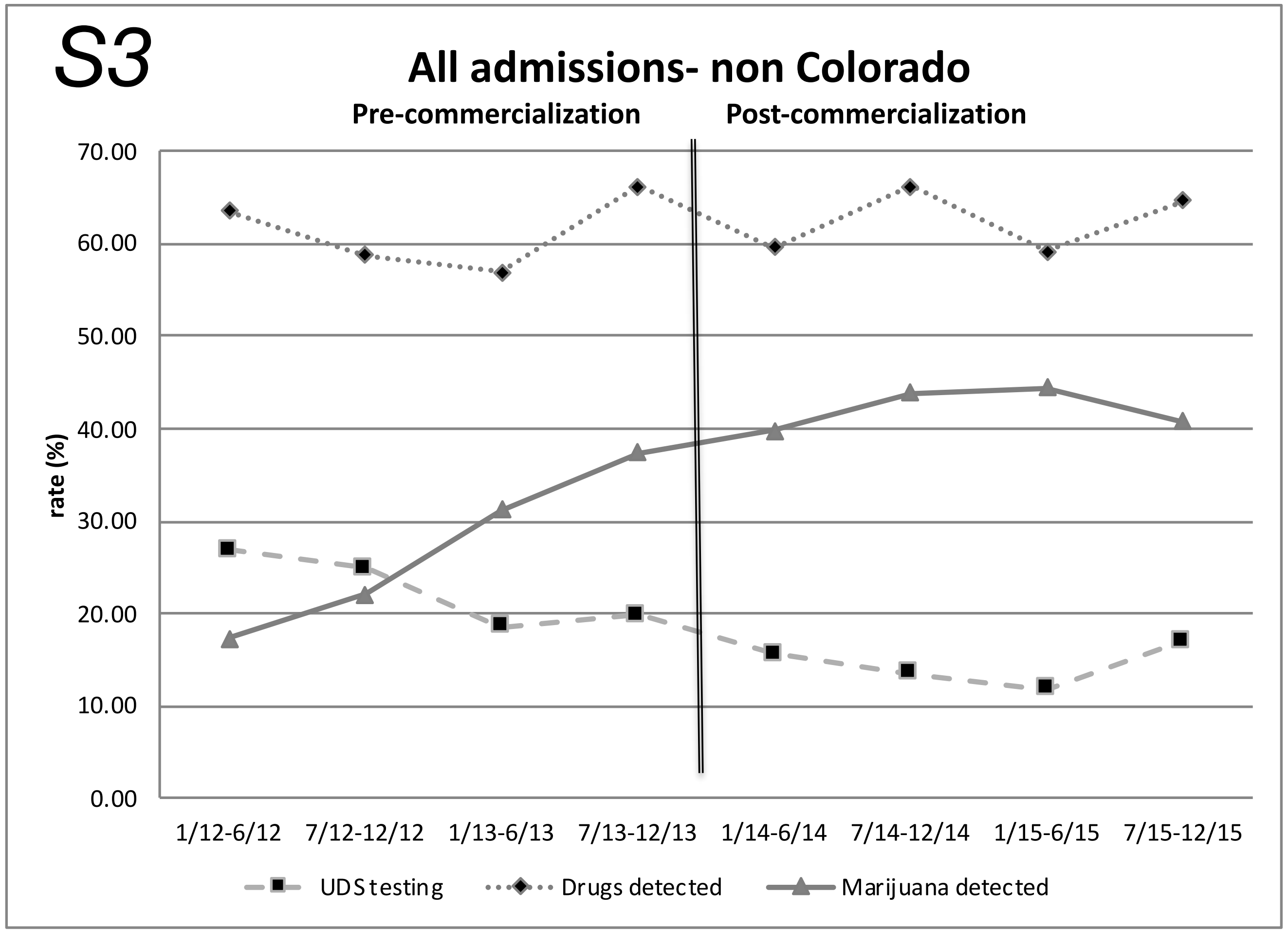
S3
All admissions- non Colorado
Pre-commercialization
Post-commercialization
rate (%)
0.00
10.00
20.00
30.00
40.00
50.00
60.00
70.00
1/12-6/12
7/12-12/12
1/13-6/13
7/13-12/13
1/14-6/14
7/14-12/14
1/15-6/15
7/15-12/15
UDS testing
Drugs detected
Marijuana detected

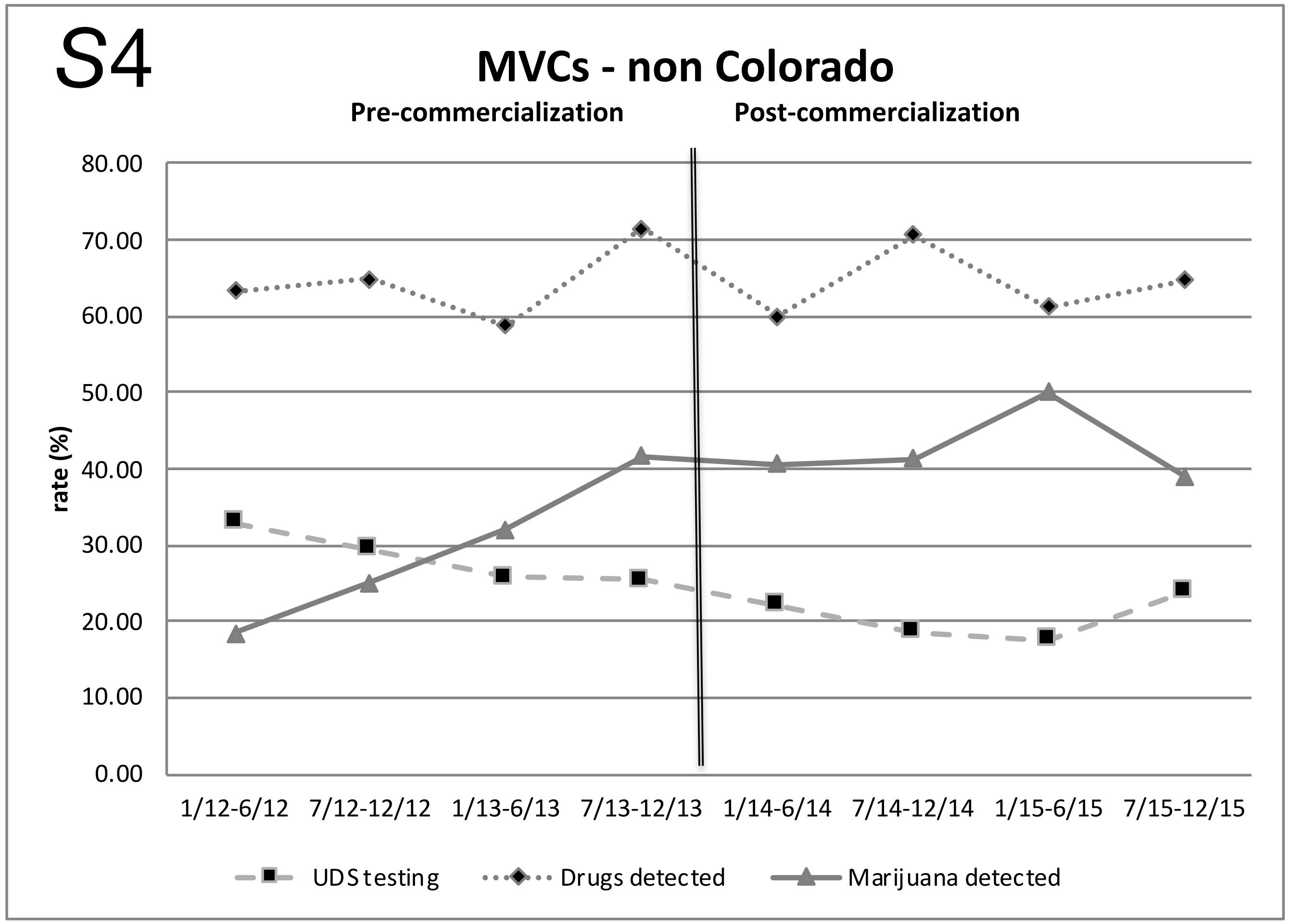
S4
MVCs - non Colorado
Pre-commercialization
Post-commercialization
rate (%)
0.00
10.00
20.00
30.00
40.00
50.00
60.00
70.00
80.00
1/12-6/12
7/12-12/12
1/13-6/13
7/13-12/13
1/14-6/14
7/14-12/14
1/15-6/15
7/15-12/15
UDS testing
Drugs detected
Marijuana detected
